# What Is an Aging-Related Disease? An Epidemiological Perspective

**DOI:** 10.1093/gerona/glac039

**Published:** 2022-02-15

**Authors:** David G Le Couteur, Janani Thillainadesan

**Affiliations:** Department of Geriatric Medicine, Concord Hospital, Sydney, New South Wales, Australia; Centre for Education and Research on Ageing, Concord Hospital, Sydney, New South Wales, Australia; Department of Geriatric Medicine, Concord Hospital, Sydney, New South Wales, Australia; Centre for Education and Research on Ageing, Concord Hospital, Sydney, New South Wales, Australia

**Keywords:** Age-related disease, Beta growth, Gompertz, Incidence

## Abstract

There are no established or standardized definitions of aging-related disease. Data from the Global Burden of Diseases, Injuries, and Risk Factors Study 2019 were used to model the relationship between age and incidence of diseases. Clustering analysis identified 4 groups of noncommunicable diseases: Group A diseases with an exponential increase in incidence with age; Group B diseases with an exponential increase in incidence that usually peaked in late life which then declined or plateaued at the oldest ages; and Groups C and D diseases with an onset in earlier life and where incidence was stable or decreased in old age. From an epidemiological perspective, Group A diseases are “aging-related diseases” because there is an exponential association between age and incidence, and the slope of the incidence curves remains positive throughout old age. These included the major noncommunicable diseases dementia, stroke, and ischemic heart disease. Whether any of the other diseases are aging-related is uncertain because their incidence either does not change or more often decreases in old age. Only biological studies can determine how the aging process contributes to any of these diseases and this may lead to a reclassification of disease on the basis of whether they are directly caused by or are in continuity with the biological changes of aging. In the absence of this mechanistic data, we propose the term “aging-related disease” should be used with precision based on epidemiological evidence.

The terms “age-related” or “aging-related” diseases are used widely and applied loosely and inconsistently to many different diseases. This is the consequence of the absence of standardized or established definitions for these terms. A core tenet of biogerontology is that aging is a major risk factor for or a direct cause of aging-related diseases. Identifying which diseases are aging-related is a fundamental step for studies of aging and the pathogenesis of diseases, or predicting which diseases might be treated or prevented by interventions that delay, or act on, aging.

Determining which diseases are aging-related will require a combination of biological, interventional, and epidemiological criteria. Epidemiological data have been used in only a few studies to explore the relationship between aging and the incidence of diseases. The underlying assumption for these studies has been that the incidence—rather than prevalence, mortality, or disease burden—of an aging-related disease continues to increase at older ages (1,2).

Here, the Global Burden of Disease database was used to (a) classify the relationships between the incidence of diseases and age across all age groups and (b) propose an epidemiological definition for “aging-related disease.” We used the term “aging-related” as suggested by Kuan et al. (2) to refer to diseases that increase with old age and therefore are more likely to be mechanistically linked to the aging process and old age. On the other hand, the term “age-related” could refer to any disease where incidence is the highest at any particular age, including childhood.

## Method

### Data Source and Variables

Publicly available data from the Global Burden of Diseases, Injuries, and Risk Factors Study (GBD) 2019 were used. The GBD study provides data on global prevalence, incidence, mortality, years of life lost, years lived with disability, and disability-adjusted life-years for 369 diseases and injuries for 204 countries (3). We extracted incidence data for all diseases, and our primary analyses included only noncommunicable diseases for which full data sets were available (Supplementary Table 1). Global data for 2019 and for both sexes were used. The entire age range was used (“0–4 years” to “95 plus”) and each 5-year age bracket was converted to a single age (eg, the 5–9 years age bracket was converted to 9) to facilitate modeling.

The effects of the sociodemographic index on the incidence of noncommunicable diseases were studied. The sociodemographic index of a country or region is a composite average of the rankings of incomes per capita, average educational attainment, and fertility rates and is strongly associated with health outcomes. The relationships between age and the incidence of communicable diseases were studied as this could provide insights into the effect of aging on the immune system and susceptibility to infections.

### Cluster Analysis

Cluster analysis was used to classify diseases with similar age versus standardized incidence curves. For each disease, data were standardized by dividing each incidence value by the area under the curve for that disease. These standardized incidence values for each age bracket were then used as the variables for agglomerative hierarchical cluster analysis. There were 20 variables relating to each 5-year age bracket up to and including 95 plus years. Analysis was performed in XLSTAT using default settings (Euclidean distance for dissimilarity, Wards method; Addinsoft 2021, XLSTAT statistical and data analysis solution, New York, NY; https://www.xlstat.com).

### Modeling and Interpretation of Incidence Curves

To visually interpret the effects of age on incidence, plots were constructed of age versus the rate of change of standardized incidence. The rate of change of incidence over each 5-year age bracket was calculated from the slope between adjacent standardized incidence values. In these graphs, positive values indicate that incidence increases with age, and negative values indicate that incidence decreases with age. For an aging-related disease, the values in this graph would be expected to be positive throughout old age.

Curves were fitted using exponential Gompertz–Makeham equation:


$$\begin{array}{l} q\left(t\right)=\gamma +\alpha e^{-\beta t} \end{array}$$

where *q*(*t*) is the incidence rate at age *t*; *t* is the age, γ is the Makeham constant that reflects factors not related to age, and β is the rate of increase of incidence with age (4).

Curves were also fitted with the beta growth function according to the equation:


$$\begin{array}{l} q\left(t\right)=\delta \left(1+\frac{\kappa -t}{\kappa -\lambda }\right){\left(\frac{t}{\kappa }\right)}^{\frac{\kappa }{\kappa -\lambda }} \end{array}$$

where δ is the size of the population at peak, κ is the age at which the population peaks, and λ is the time of the inflection point of the growth curve. For beta growth curves, growth is maximum at the inflexion point of the curve; and this falls to zero at the age when incidence is maximum (5).

The curves for age versus incidence were fitted using the nonlinear regression function and plotted in XLSTAT at the default settings (500 iterations, convergence 0.00001).

## Results

### Clustering

There were 92 noncommunicable diseases included in the analysis. The number of clusters selected was 4 on the basis of the elbow method, appearance of dendrograms, the number of diseases per group, and visual examination of the diseases and their incidence curves in each group (Supplementary Figure 1A and B). Truncation to 4 groups had a cophenetic correlation of 0.68. The diseases in each of the 4 groups are given in Table 1.

**Table 1.** Four Groups of Noncommunicable Diseases Identified Using Agglomerative Hierarchical Clustering

Group A (*n* = 22)	Group B (*n* = 29)	Group C (*n* = 20)	Group D (*n* = 21)
Alzheimer’s disease and other dementias Bladder cancer Cardiomyopathy and myocarditis Chronic lymphoid leukemia Chronic myeloid leukemia Chronic obstructive pulmonary disease Colon and rectum cancer Gallbladder and biliary tract cancer Hodgkin lymphoma Intracerebral hemorrhage Ischemic heart disease Ischemic stroke Malignant skin melanoma Myocarditis Non-Hodgkin lymphoma Nonmelanoma skin cancer (basal-cell carcinoma) Nonmelanoma skin cancer (squamous-cell carcinoma) Pancreatic cancer Paralytic ileus and intestinal obstruction Stroke Subarachnoid hemorrhage Vascular intestinal disorders	Asbestosis Atrial fibrillation and flutter Brain and central nervous system cancer Breast cancer Chronic kidney disease Chronic kidney disease due to type 2 diabetes mellitus Chronic kidney disease due to hypertension Coal workers pneumoconiosis Esophageal cancer Gout Interstitial lung disease and pulmonary sarcoidosis Kidney cancer Larynx cancer Lip and oral cavity cancer Liver cancer Liver cancer due to NASH Mesothelioma Motor neuron disease Multiple myeloma Nonrheumatic calcific aortic valve disease Ovarian cancer Pancreatitis Parkinson’s disease Peptic ulcer disease Prostate cancer Stomach cancer Thyroid cancer Tracheal, bronchus, and lung cancer Uterine cancer	Acute lymphoid leukemia Benign and in situ intestinal neoplasms Benign prostatic hyperplasia Cervical cancer Contact dermatitis Depressive disorders Dermatitis Type 2 diabetes mellitus Gastritis and duodenitis Gastroesophageal reflux disease Inflammatory bowel disease Inguinal, femoral, and abdominal hernia Major depressive disorder Nasopharynx cancer Nonrheumatic degenerative mitral valve disease Osteoarthritis Pneumoconiosis Rheumatoid arthritis Silicosis Urolithiasis	Acute glomerulonephritis Alopecia areata Anxiety disorders Appendicitis Asthma Atopic dermatitis Benign and in situ cervical and uterine neoplasms Bipolar disorder Chronic kidney disease due to type 1 diabetes mellitus Chronic kidney disease due to glomerulonephritis Cirrhosis and other chronic liver diseases Cirrhosis and other chronic liver diseases due to NAFLD Type 1 diabetes mellitus Idiopathic epilepsy Migraine Multiple sclerosis Psoriasis Schizophrenia Seborrheic dermatitis Testicular cancer Uterine fibroids

*Note:* NASH = non-alcoholic steatohepatitis; NAFLD = non-alcoholic fatty liver disease.

There were 47 communicable diseases that clustered into 4 groups (Supplementary Table 2). There was 1 group with 7 diseases where there was an increase in incidence in old age. These diseases were mostly broad diagnostic categories (cellulitis, endocarditis, enteric infections, fungal skin diseases, and lower respiratory infections) rather than specific microbial infections.

### Incidence and Rate of Change of Incidence Curves

**Figure 1.**
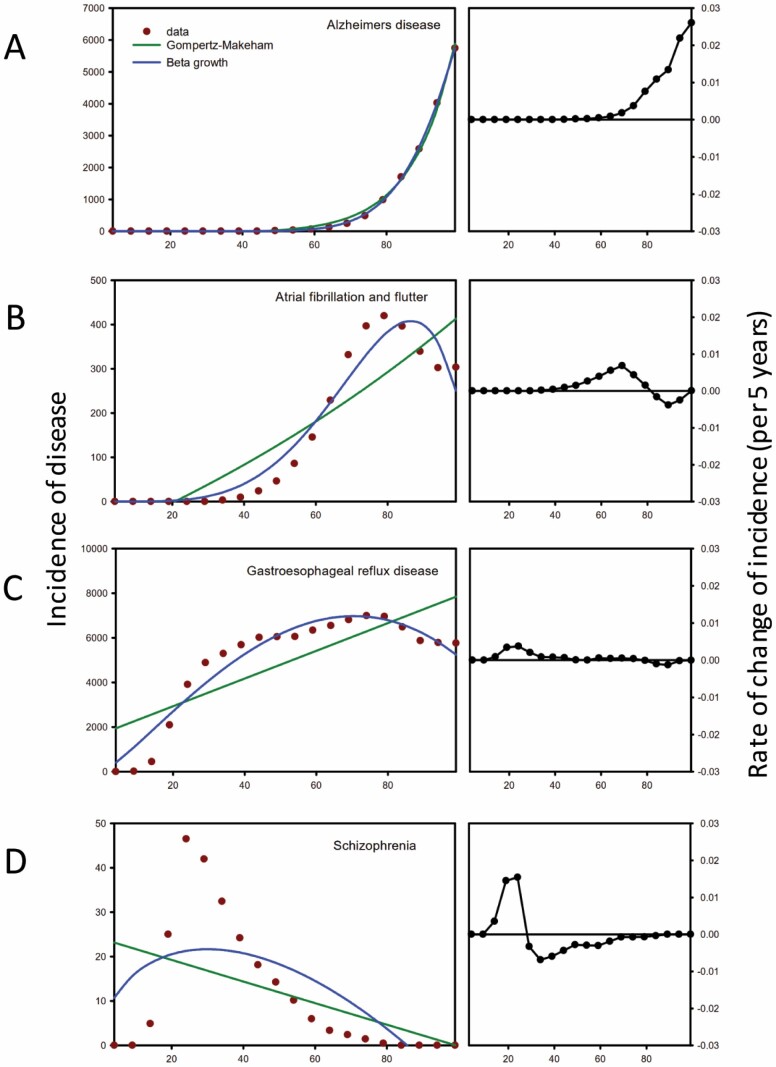
Examples of the 4 types of relationships between age and incidence of disease from the Global Burden of Disease database (GBD) for (**A**) Group A: Alzheimer’s disease and other dementias; (**B**) Group B: atrial fibrillation and flutter; (**C**) Group C: gastroesophageal reflux disease; and (**D**) Group D: schizophrenia. The curves have been fitted with the Gompertz–Makeham formula (green line) and beta growth curve (blue line). The curves on the right show the relationship between age and the rate of change of incidence. In these graphs, positive values indicate that incidence is increasing with age and negative values indicate that incidence is decreasing.

Examples of incidence curves from each group are shown in Figure 1, and curves for all individual diseases, sorted into Groups A–D by cluster analysis, are shown in Supplementary Figures 2–5. Overall, the curves within each group were similar as follows:

- Group A included only diseases (*n* = 22) where there was an exponential increase in incidence in old age. The curve for the rate of change of incidence was positive for all age brackets beyond the ages of 50–60 years.
- Group B included diseases (*n* = 24) where incidence increased exponentially until ages between 60 and 80 years, then plateaued or decreased. The curves for the rate of change of incidence were initially positive, then negative, in old age for the curves that plateaued or declined at an old age. There were also a few diseases in this group where incidence increased monotonically but irregularly (breast cancer, coal workers pneumoconiosis, ovarian cancer, pancreatitis, and peptic ulcer disease), and the rate of change of incidence was positive.
- Group C included diseases (*n* = 20) that reached peak incidence at about 50–70 years, then plateaued or decreased substantially at older ages. The curves for the rate of change of incidence in old age were either negative or zero in older ages. The difference between Groups B and C diseases is primarily quantitative related to the age of peak incidence and the extent that the curves declined.
- Group D included diseases (*n* = 21) where incidences were the highest in childhood or peaked in early adult life, then decreased substantially usually to zero.

**Figure 2.**
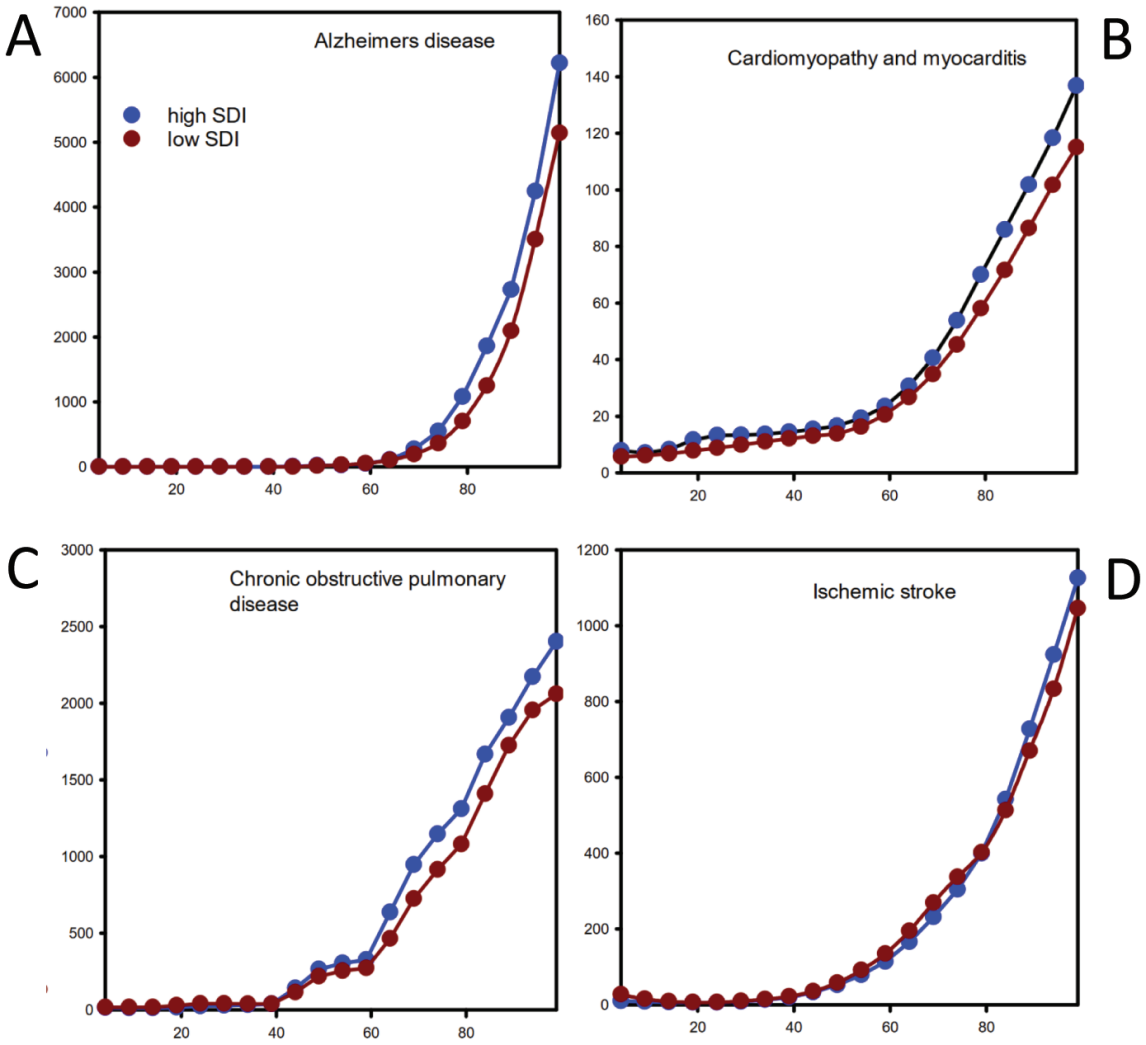
The relationship between the sociodemographic index (SDI) on the relationship between age and incidence of disease for Group A diseases where sociodemographic index has minimal affect on incidence: (**A**) Alzheimer’s disease, (**B**) cardiomyopathy and myocarditis, (**C**) chronic obstructive pulmonary disease, and (**D**) ischemic stroke. *Note:* Blue symbols = high sociodemographic index; dark red symbols = low sociodemographic index.

The incidence curves from regions with low versus high sociodemographic indices were compared (Figure 2 and Supplementary Figure 6). There were some curves that overlapped closely and they included diseases from each group (Table 2).

**Table 2.** Diseases Where the Relationship Between Age and Incidence Was Similar in Regions With a Low or High Sociodemographic Index

Group A	Group B	Group C	Group D
Alzheimer’s disease Cardiomyopathy and myocarditis Chronic obstructive pulmonary disease Ischemic stroke	Lip and oral cavity cancer	Benign prostatic hyperplasia Rheumatoid arthritis	Asthma Bipolar disorder Idiopathic epilepsy Seborrheic dermatitis

### Curve Modeling

The fitted curves are shown for each disease in Supplementary Figures 2–5. Overall, the Gompertz model fitted the curves with an exponential increase in incidence in Group A, while the beta growth model fitted curves in Groups A–C that had an increase in incidence followed by a plateau or decline in incidence. For many of the exponential curves, the beta growth model could not return plausible values for the age at peak incidence or inflexion of the curve, because, if present they were beyond the age boundaries. The results from these curves were excluded from grouped data below.

Analysis was restricted to those diseases where the goodness of fit was reasonable (*R*^2^ > 0.90) and the curves either had a monotonic increase for the Gompertz model and/or had an increasing incidence with a late-life decline or plateau in the beta growth model. For Group A, the Gompertz model yielded a value for β of 0.043 ± 0.013 (*n* = 22) and for Group B was 0.020 ± 0.009 (*n* = 12). This means that the incidence of diseases doubled every 16 years for diseases in Group A and every 35 years in Group B.

The beta growth model yielded values for κ in Groups A–D of 135 ± 69, 92 ± 11, 72 ± 6, and 24 ± 20 years, respectively. The variable κ refers to the age at which incidence is at its peak, and the underlying process driving change in the rate of incidence is zero. The model yielded values for λ in Groups A–D of 111 ± 55, 71 ± 7, 29 ± 20, and −65 ± 90 years. This refers to the age at which inflexion of the curve occurs during the initial phase where incidence is increasing, and the underlying process driving change in incidence is maximal. These values for λ and κ also indicate the order of the groups in terms of age (Group A is the oldest, through to Group D which is the youngest).

## Discussion

Aging is often stated to be a major risk factor for noncommunicable and chronic diseases, and this is an important motivation for studying aging biology and developing interventions to delay aging (6–10). Ultimately the proof of the role aging plays in the pathogenesis of any disease will depend upon biomedical research showing that the biological changes of aging are necessary for the development of disease and that interventions that delay aging also delay disease. Even so, epidemiological data can also provide insights into the strength and type of the associations between aging and disease, and which diseases may be the most coherently designated as “aging-related”. Similar to other studies, we investigated the incidence of the disease on the assumption that, or by definition, an aging-related disease is one where the incidence of the disease continues to increase into old age. On the other hand, the relationship between age and prevalence will indicate whether a disease is common in older people but this is a function of the age of onset of disease (incidence) as well as the duration of survival with the disease and the size of each age group.

Diseases could be clustered into 4 groups, based on the incidence curves. The first group (Group A) of diseases was those that had an exponential association between age and incidence, with the increase in incidence continuing into the oldest age groups. This included major communicable diseases (ischemic heart disease, stroke and intracerebral hemorrhage, Alzheimer’s disease, chronic obstructive pulmonary disease) and some cancers. The curves were effectively fitted by a simple Gompertz–Makeham model. This equation was originally formulated on the assumption that the force of mortality generated by aging increases exponentially with age, and hence mortality also increases exponentially. A similar assumption can be applied to the incidence of disease. For Group A diseases, the average β from the Gompertz–Makeham formula was 0.043, which is about half the value for human mortality (β = 0.087, Supplementary Figure 7). This can be interpreted as that the incidence of these diseases doubles every 18 years, compared with mortality that doubles every 8 years. The finding that many different types of diseases have an exponential relationship with age makes it more likely that aging is directly associated with these diseases rather than being a confounding factor. Moreover, the epidemiological association between these diseases and aging fulfill at least some of the Bradford Hill criteria for causality (Table 3), acknowledging the limitations of this approach (11). Regardless of whether the biological changes of aging are mechanistically linked to these Group A diseases, the epidemiological concept that aging-related diseases be restricted to those where incidence increases with chronological age is axiomatic. Conversely, diseases where incidence decreases or is unchanged in old age are not aging-related, at least from the epidemiological perspective. Epidemiological associations do not prove causation; therefore, biological studies will be the final judge as to whether biological aging changes have a mechanistic role in any of these diseases.

**Table 3.** Bradford Hill Causality Criteria Applied to Those Diseases With an Exponential Association Between Age and Incidence

Criteria	Applied to Aging and Diseases With an Exponential Association Between Age and Incidence (Type A)
Strength	Yes. The goodness of fit parameters are very high
Consistency	Yes. The exponential association occurs in multiple diseases and has been reported elsewhere using different databases
Temporality	Likely. Type A diseases are rare at young ages, and the incidence continues to increase at older ages
Biological gradient	Yes. A biological gradient is inherent in the exponential association
Specificity of association	Likely. Aging is universal so specificity cannot be determined. The specificity might be strengthened if biological aging is found to be greater in those with Type A diseases compared to other diseases without an exponential association
Biological plausibility	Yes. Based on studies finding similarities in the biological mechanisms for aging and mechanisms for Type A diseases
Coherence	Yes. All these diseases progress with age
Experiment	No. However, caloric restriction studies in animals indicate a reduction in some diseases
Analogy	Possible. If aging is considered a physiological part of life, then there may be an analogy with the relationship between sexual maturation and diseases of puberty

The incidence of the Group A diseases varied substantially from relatively low incidence (melanoma, subarachnoid hemorrhage) to high incidence (Alzheimer’s disease, ischemic heart disease). The question then arises, how such variability might occur given aging is universal? Linked to this issue is contingency, whether any effects of aging biology on the incidence of aging-related diseases are contingent on the presence of other risk factors or mechanisms that might be present in some populations but not others. For example, the incidence of skin cancers (melanoma, basal cell carcinoma, squamous cell carcinoma) increased with old age, and sun exposure is a major risk factor. It could be hypothesized that sun exposure either accelerates skin aging or acts synergistically with aging to produce skin cancers. To investigate contingency, the effects of the sociodemographic index were studied, on the assumption that this parameter reflects a range of environmental health-related factors that might influence the relationship between aging on incidence. There were 4 Group A diseases (Alzheimer’s disease, cardiomyopathy and myocarditis, chronic obstructive pulmonary disease, and ischemic heart disease), where the relationship between age and incidence was not influenced by the sociodemographic incidence. This might provide evidence that intrinsic aging biology is likely to be involved in the pathogenesis of these 4 diseases. However, the relationships between age and the incidence of some diseases from the other groups were not influenced by the sociodemographic index. It should be noted that there is a stochastic component to aging, and differences in the incidences of aging-related disease might be simply a result of chance.

There have been other epidemiological studies that have attempted to identify aging-related diseases. Kuan et al. (2) clustered diseases from an English primary practice database into 9 groups on the basis of median age of onset of disease. Diseases from clusters with an older age of onset that also could be fitted with modified Gompertz–Makeham formula were considered to be aging-related. They identified 210 age-related diseases. They excluded people older than 85 years because of concern that the incidence in very old people can be influenced by artifacts (eg, reduced rates of cancer screening). They also modified the Gompertz–Makeham with a polynomial term to fit those diseases with a late-life plateau or decline in incidence. By comparison, we included all ages up to “95 plus” and categorized those diseases with a late-life plateau or decline into a separate group. Chang et al. (1) used the Global Burden of Disease (2017) database to study “aging burden” defined by those diseases where the incidence increased quadratically with age. They identified 92 age-related diseases although this included injuries, congenital disorders, and some communicable diseases. All the diseases we identified as age-related were included in their grouping of age-related diseases. Zenin et al. (12) in a study of the UK Biobank concluded that the incidence of 7 age-related diseases (cancer, heart failure, chronic obstructive pulmonary disease, myocardial infarction, stroke, dementia, and diabetes) had the same doubling time as Gompertz mortality of about 8 years. Donertas et al. (13) used clustering to identify groups of diseases with different ages of onset in the UK Biobank. Their Genome-Wide Association Study showed that the clusters of disease shared similar gene variants, and that these variants were often involved in aging pathways. The authors acknowledged that their study lacked data from people older than 65 years, and the age of onset of the diseases in the clusters was 20–40 years.

The second group of diseases was those with a late-life decline or plateau in incidence, which occurred over the age of about 80 years (most Group B diseases). This group of diseases included many cancers along with conditions usually described as aging-related (Parkinson’s disease, atrial fibrillation, gout). This type of curve with a late-life decline has been identified previously with mortality (14,15). Several hypotheses have been proposed for late-life decline in mortality including that there is a cohort of healthy survivors with delayed mortality or frail people with earlier mortality, or that this is simply the result of errors in data collection (16). Late-life decline in the incidence of some diseases was found in 2 US databases, where it was attributed to either effects of selection (frail older people do not survive to old age), artifact (underdiagnosis in very old people), or a change in the susceptibility of very old people to death and disease (17). In another study of US male physicians, the incidence of cardiovascular disease continued to increase, while that of cancer peaked between 80 and 89 years, then underwent a late-life decline. It was concluded that this might be secondary to reduced cancer screening in the oldest males (18). In order to avoid this issue in their study, Kuan et al. (2) excluded data in people older than the age of 85 years. An alternative approach was taken by Belikov (19) who used the Erlang distribution to fit the incidence data of cancers from the CDC WONDER database. Of the 19 cancers in common with our analysis, 58% had late-life decline or plateau and 42% were exponential, although data beyond 85 years was not provided. This compared with 74% in our database that had late-life decline and 26% were exponential. There was reasonable agreement between the 2 studies, the difference being our data was extended to 95 plus years, allowing late-life decline to become apparent after 85 years. Belikov used the curving fitting to model the number of hits to produce cancer, but did not discuss mechanisms for the late-life decline.

The finding that the beta growth formula fitted the curves may provide some insights into the pathogenesis of these diseases that are not related to artifacts or errors. Growth curves are modeled on the assumption that the presence of a peak in a growth curve indicates that there are 2 processes—the first that accelerates growth and the second decelerates growth. In the case of beta growth curves, the first process has its peak effect at the inflexion point of the curve, then declines to zero at the age when growth reaches its peak. In Group B diseases, such a process would therefore peak at age 72 ± 6 years then decline to zero at 92 ± 11 years. Given these 2 ages are old, it might be reasonable to assume that this process is aging, and therefore these diseases are age-related. However, this does not provide any explanation for the second decelerating process (and why incidence declines), which must be present in Type B diseases, but not Type A diseases.

Another possibility is that the second process that decelerates the incidence of some diseases in very old age is in fact aging. It is of note that many of these Type B diseases were cancers. It has been proposed that age-related immunosenescence leads to changes, such as increased NK cells, that could reduce the growth of tumors and reduce cancer incidence in very old people (20). Other hallmarks of aging such as telomere attrition and cellular senescence may also reduce oncogenesis (21,22), a type of reverse antagonistic pleiotropy, or “adaptive senectitude” (23), where processes that are harmful or have no benefit at younger ages become beneficial at older ages. It follows that there would be a paradox where Type A diseases are “caused” by aging and are truly “age-related” diseases, while Type B diseases are those in which aging biology reduces their incidence in very old people. It is of interest that frailty in humans, which we consider to be a multisystem aging syndrome (24), increases the risk of cardiovascular disease (Type A disease) but not cancers (many of which are Type B diseases) (25). An alternative, albeit overlapping hypothesis is that Type B diseases are caused by aging, but there are many biological processes that contribute to aging (the 9 Hallmarks of Aging), and these processes may have different trajectories over different timescales in different tissues. For cancers with Type B incidence curves, we could hypothesize that the age-related increase in incidence until 70–80 years is a consequence of age-related changes in epigenetics and genome instability, but beyond that age, cellular senescence and telomere attrition accelerate and become the dominant aging change, with the outcome of reducing the incidence of cancer.

The third group of diseases was those where incidence reached a peak in midlife/early late life and decreased toward zero in later life (Group C). Aging biology is unlikely to be a major driver of the pathogenesis of these diseases because incidence decreases substantially, rather than increases, with age beyond middle age. Interestingly, this included some diseases that are typically described as age-related: type 2 diabetes mellitus, osteoarthritis, and benign prostatic hypertrophy. Although these diseases are prevalent in older people, the incidence curves from the GBD database indicate that these diseases are unlikely to be caused by aging.

The final group included diseases where incidence was greater in younger age groups (Group D). These curves had a variety of shapes including biphasic curves and curves skewed to the left. This group includes diseases that are well established to occur at younger ages and rarely have their onset in older people such as asthma, schizophrenia, anxiety, and type 1 diabetes mellitus. As the focus of our study is aging, we excluded diseases that were congenital or maternal which would be likely to populate this group.

There are limitations to consider when interpreting these findings. International observational studies of the incidence and prevalence of diseases can be influenced by several types of bias including reporting biases (eg, underreporting of diseases and differences in missing data might vary depending on the wealth of the region) and cohort effects (eg, some age groups may have different exposure to risk factors and interventions to prevent diseases). The relationships between age and incidence were not adjusted for potential confounders, such as sex, genetic predisposition, socioeconomic status, environmental exposures, time period, and birth cohort effects. These may influence the aging process as well as disease susceptibility. As with all epidemiological studies of risk factors for diseases, the conclusions will become more robust if similar findings are subsequently reported in other epidemiological data sets. Although epidemiological data have a limited role in characterizing mechanisms, we have speculated how the biological changes of aging might contribute to Group A and B diseases.

In conclusion, care is needed when stating that aging is a major risk factor or cause of many diseases, or that many diseases are “age-related” and “aging-related”. From an epidemiological viewpoint, it is justifiable to classify as “aging-related” those diseases where incidence continues to increase throughout old age, and even more so when this is not influenced by other environmental factors. Here this included Alzheimer’s disease, cardiomyopathy and myocarditis, chronic obstructive pulmonary disease, and ischemic heart disease. For other diseases where the incidence was either constant or decreased with old age, the term aging-related does not appear to be appropriate. Some diseases commonly considered to be aging-related (osteoarthritis, benign prostatic hypertrophy, type 2 diabetes mellitus) were clustered into groups of diseases usually considered to be diseases of earlier life. The association between aging and disease is a major focus of biogerontology. We should establish definitions and criteria for aging-related diseases, based on epidemiological concepts such as those proposed here, and biological criteria.

## Supplementary Material

glac039_suppl_Supplementary_Figures

glac039_suppl_Supplementary_Tables
