# Supplementary material for: What Is an Aging-Related Disease? An Epidemiological Perspective
Source: J Gerontol A Biol Sci Med Sci. 2022 Feb 15;77(11):2168–74. doi: 10.1093/gerona/glac039 (PMC9678203; doi:10.1093/gerona/glac039)
Supplement: glac039_suppl_Supplementary_Figures [file glac039_suppl_supplementary_figures.pptx]

## Slide 1
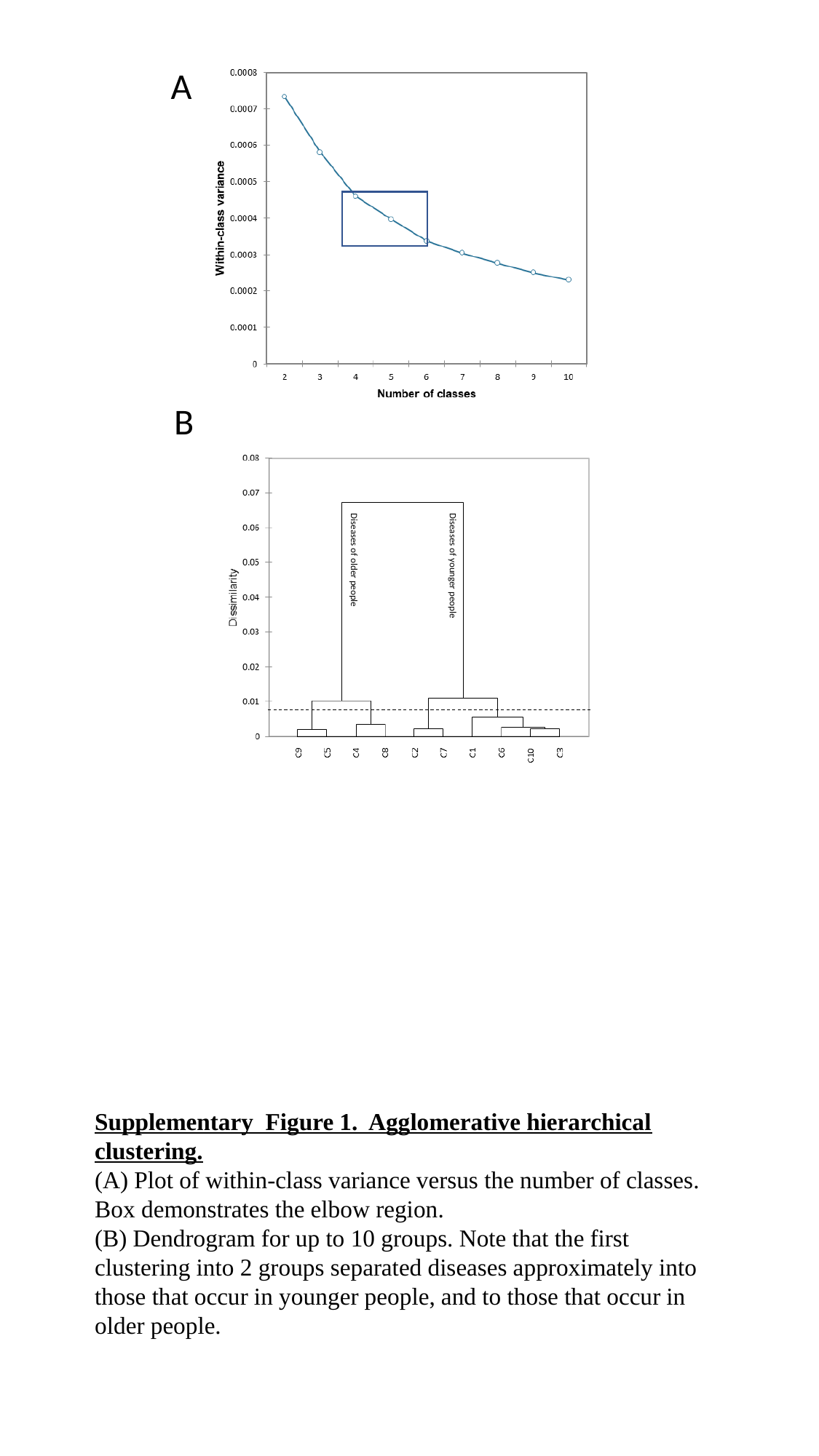

A
B
Diseases of older people
Diseases of younger people
Supplementary Figure 1. Agglomerative hierarchical clustering.
(A) Plot of within-class variance versus the number of classes. Box demonstrates the elbow region.
(B) Dendrogram for up to 10 groups. Note that the first clustering into 2 groups separated diseases approximately into those that occur in younger people, and to those that occur in older people.

## Slide 2
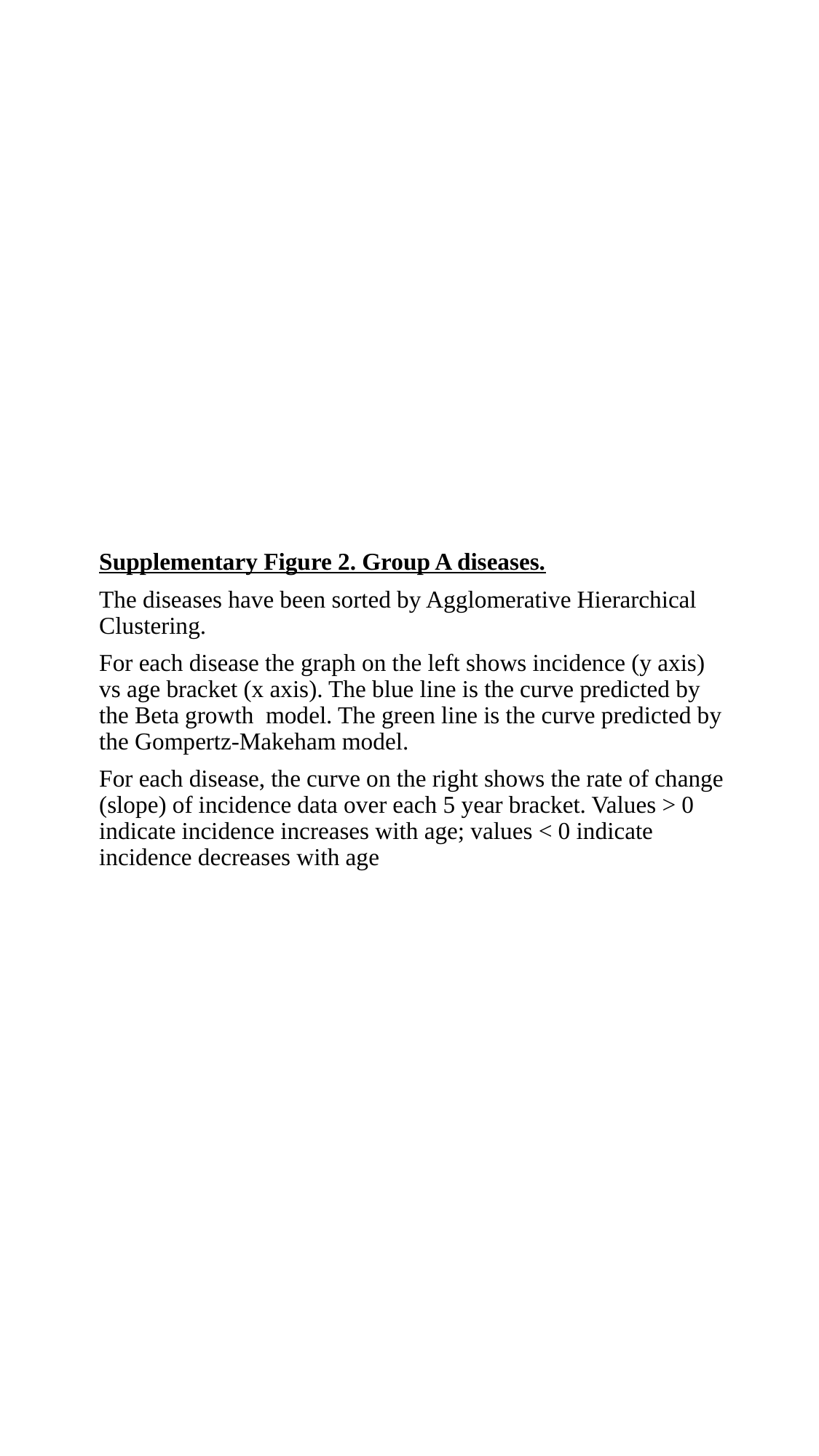

Supplementary Figure 2. Group A diseases.
The diseases have been sorted by Agglomerative Hierarchical Clustering.
For each disease the graph on the left shows incidence (y axis) vs age bracket (x axis). The blue line is the curve predicted by the Beta growth model. The green line is the curve predicted by the Gompertz-Makeham model.
For each disease, the curve on the right shows the rate of change (slope) of incidence data over each 5 year bracket. Values > 0 indicate incidence increases with age; values < 0 indicate incidence decreases with age

## Slide 3
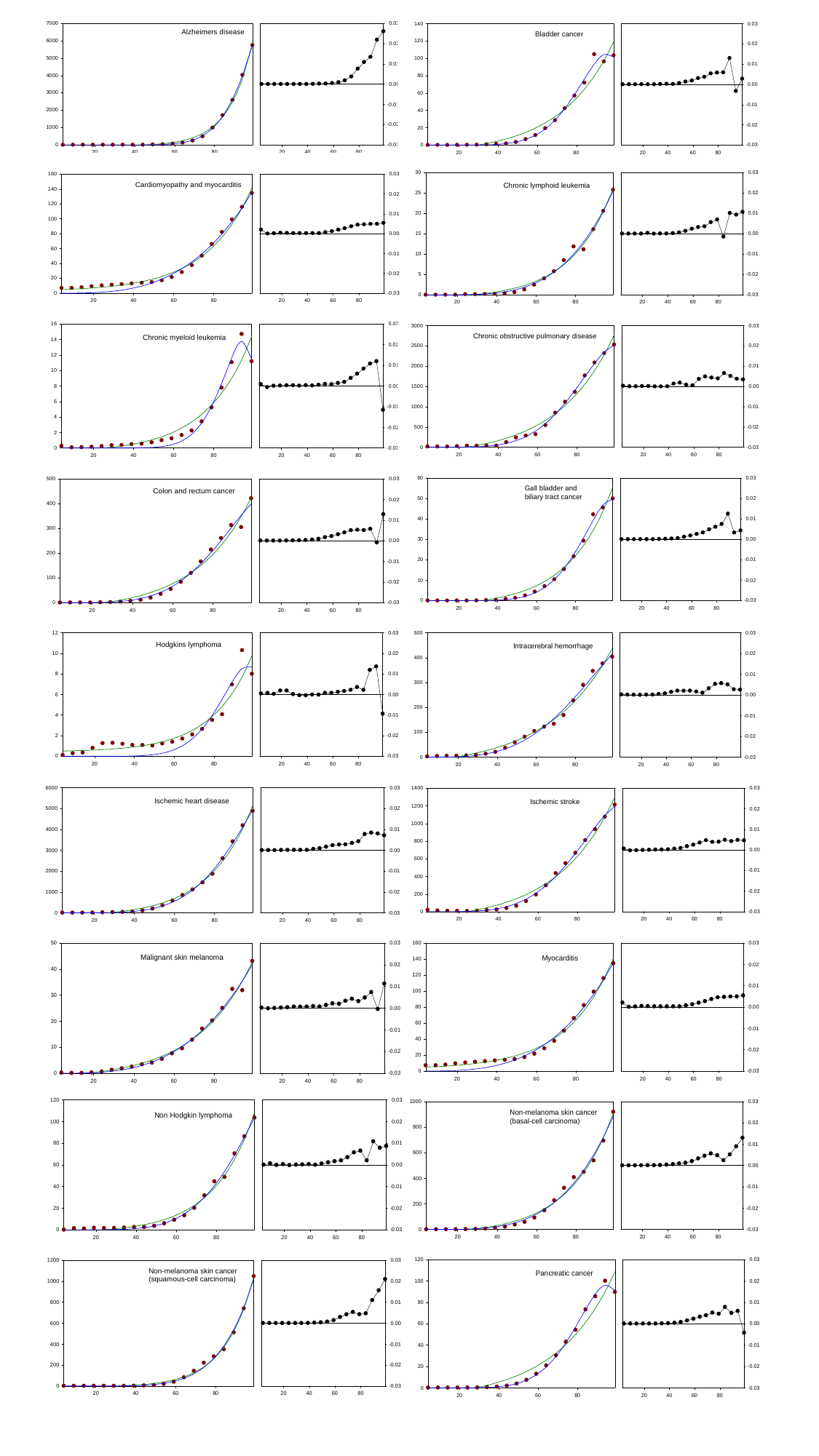

## Slide 4
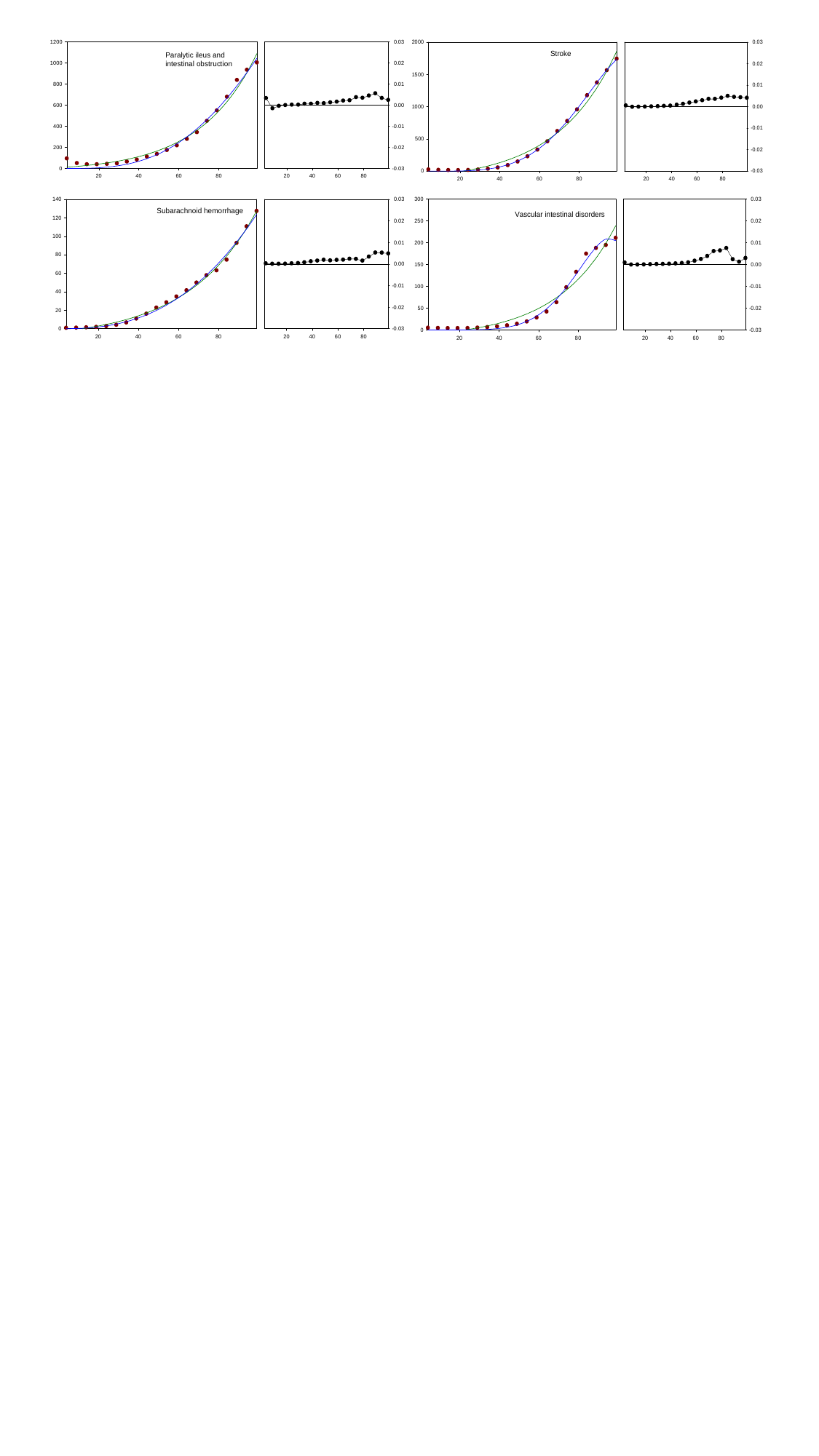

## Slide 5
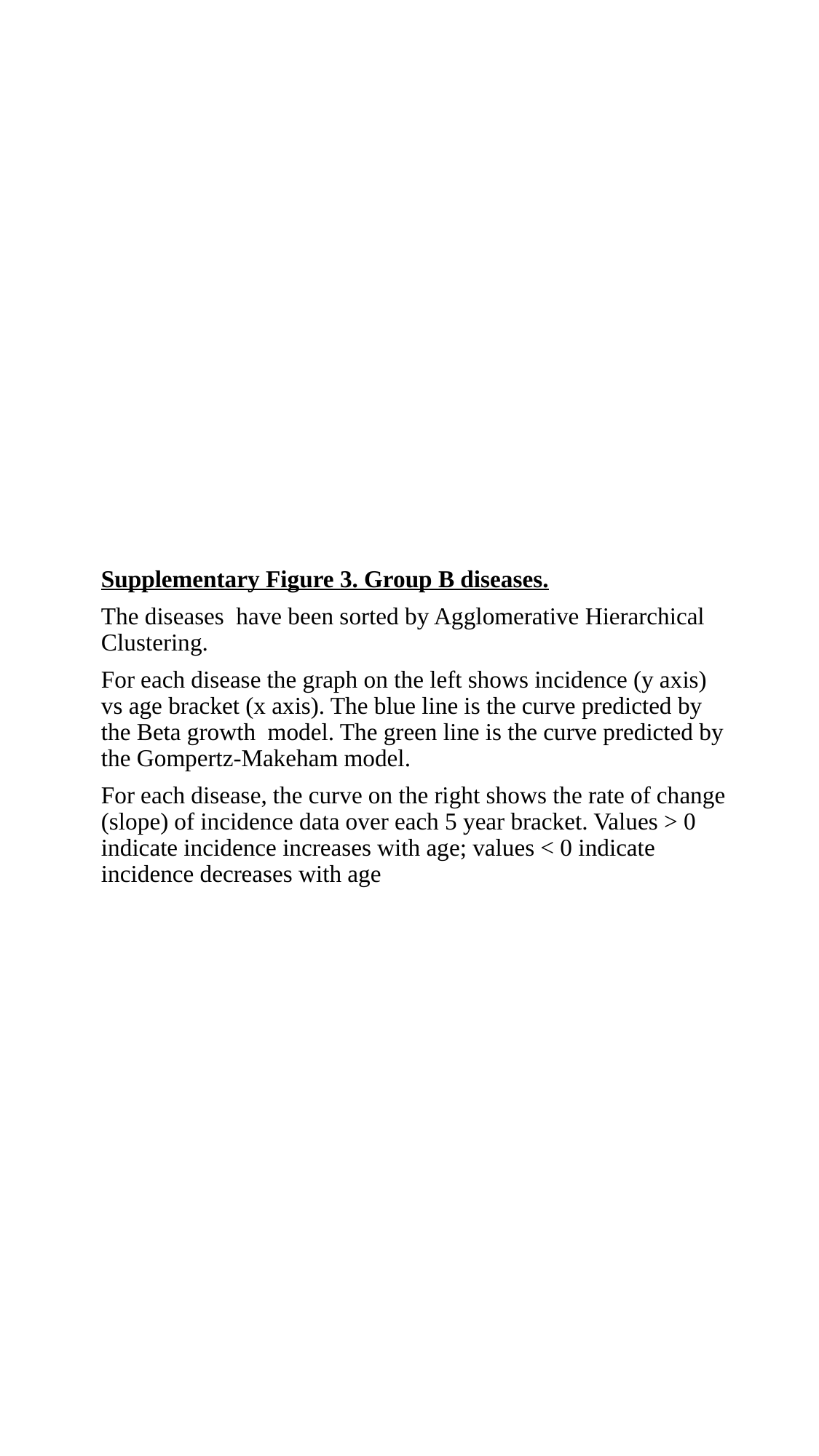

Supplementary Figure 3. Group B diseases.
The diseases have been sorted by Agglomerative Hierarchical Clustering.
For each disease the graph on the left shows incidence (y axis) vs age bracket (x axis). The blue line is the curve predicted by the Beta growth model. The green line is the curve predicted by the Gompertz-Makeham model.
For each disease, the curve on the right shows the rate of change (slope) of incidence data over each 5 year bracket. Values > 0 indicate incidence increases with age; values < 0 indicate incidence decreases with age

## Slide 6
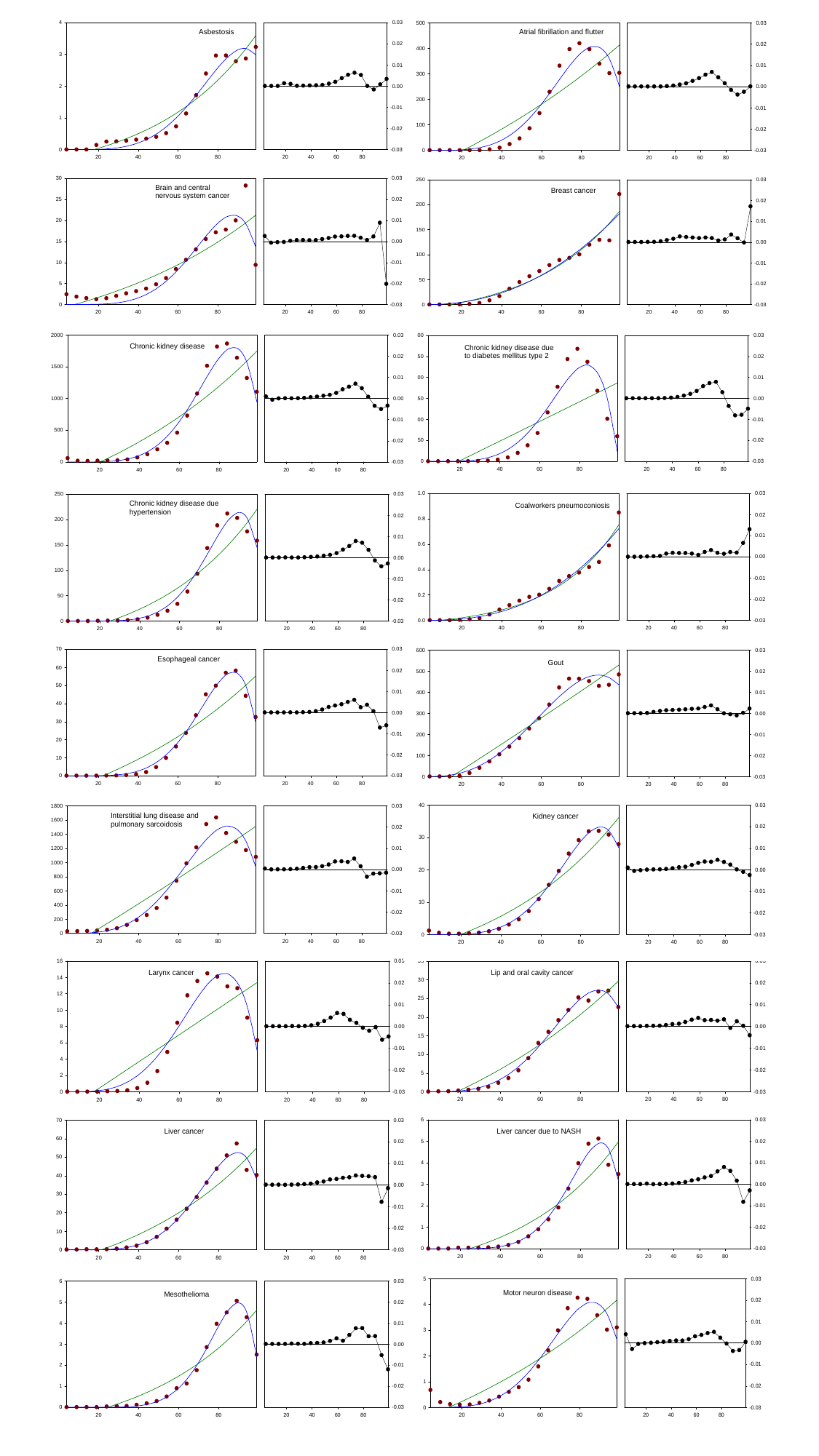

## Slide 7
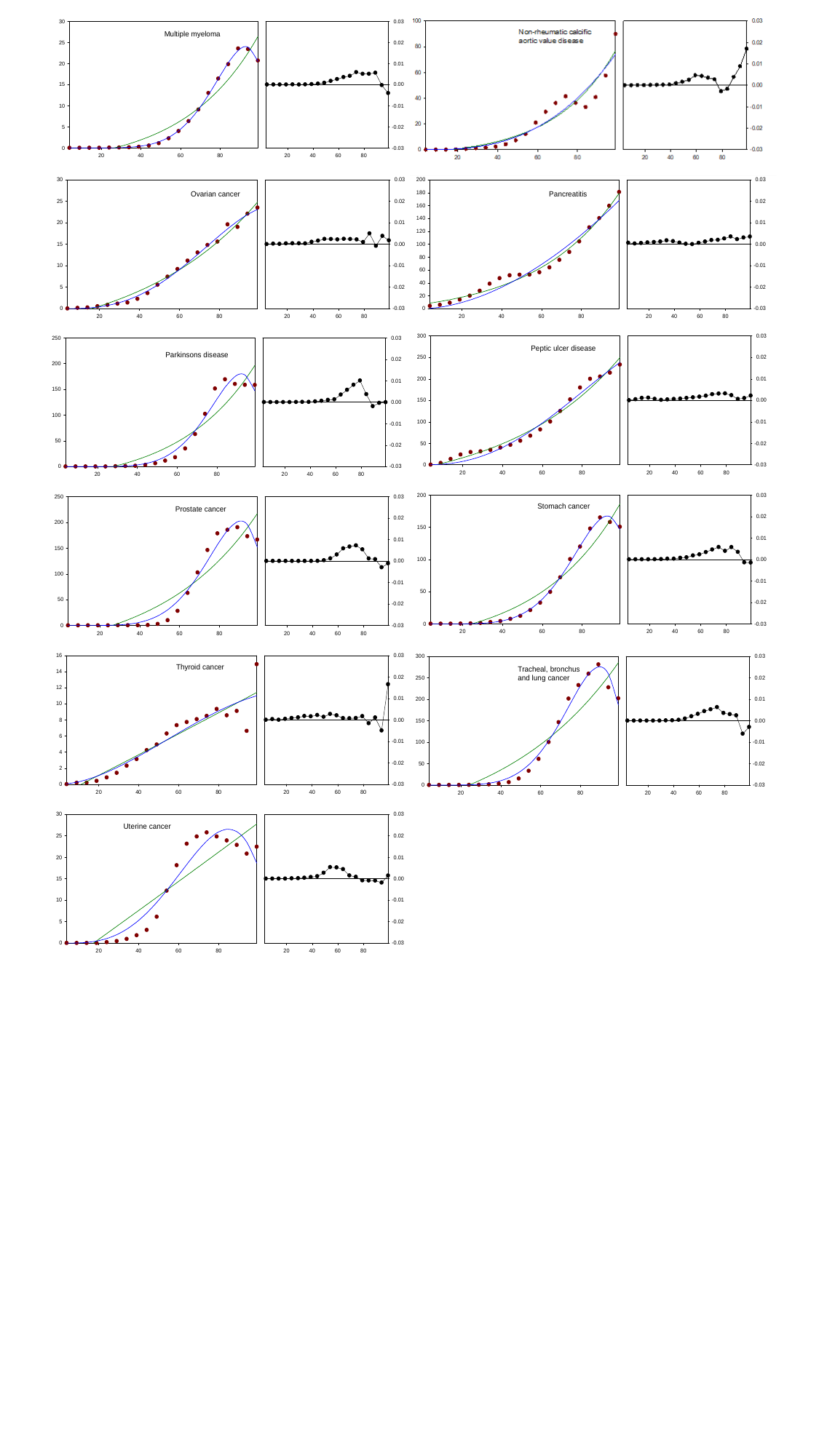

## Slide 8
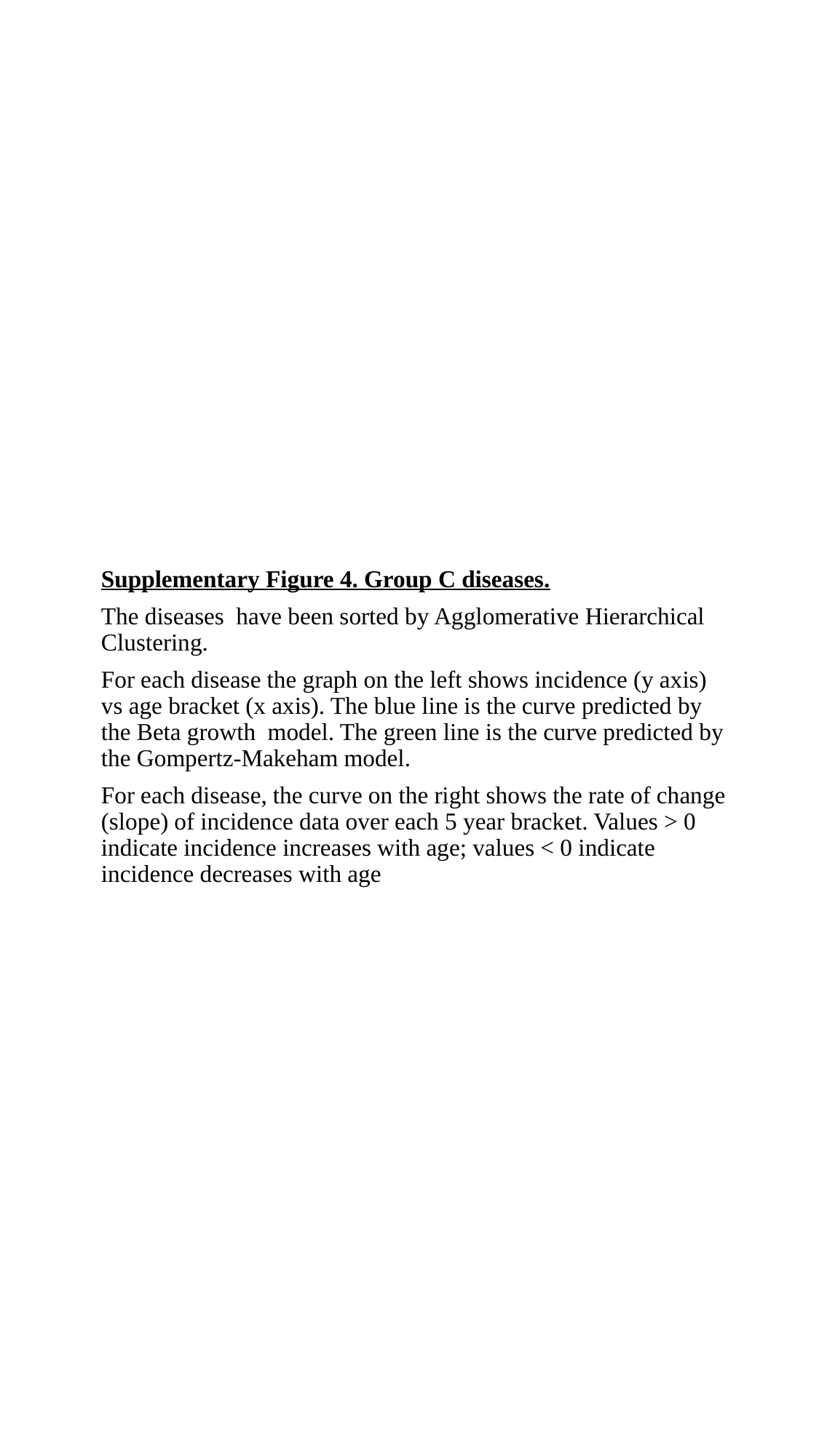

Supplementary Figure 4. Group C diseases.
The diseases have been sorted by Agglomerative Hierarchical Clustering.
For each disease the graph on the left shows incidence (y axis) vs age bracket (x axis). The blue line is the curve predicted by the Beta growth model. The green line is the curve predicted by the Gompertz-Makeham model.
For each disease, the curve on the right shows the rate of change (slope) of incidence data over each 5 year bracket. Values > 0 indicate incidence increases with age; values < 0 indicate incidence decreases with age

## Slide 9
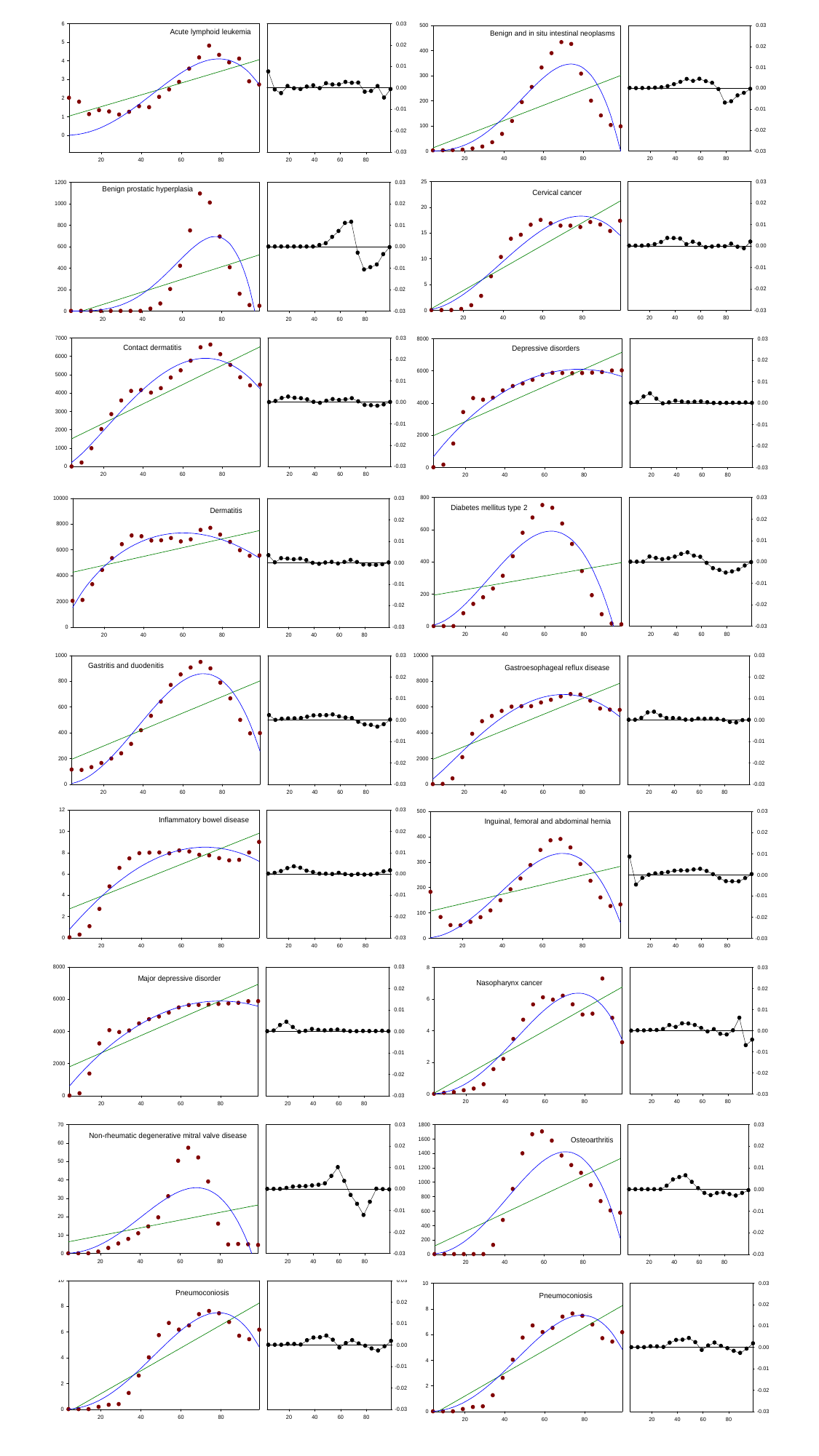

## Slide 10
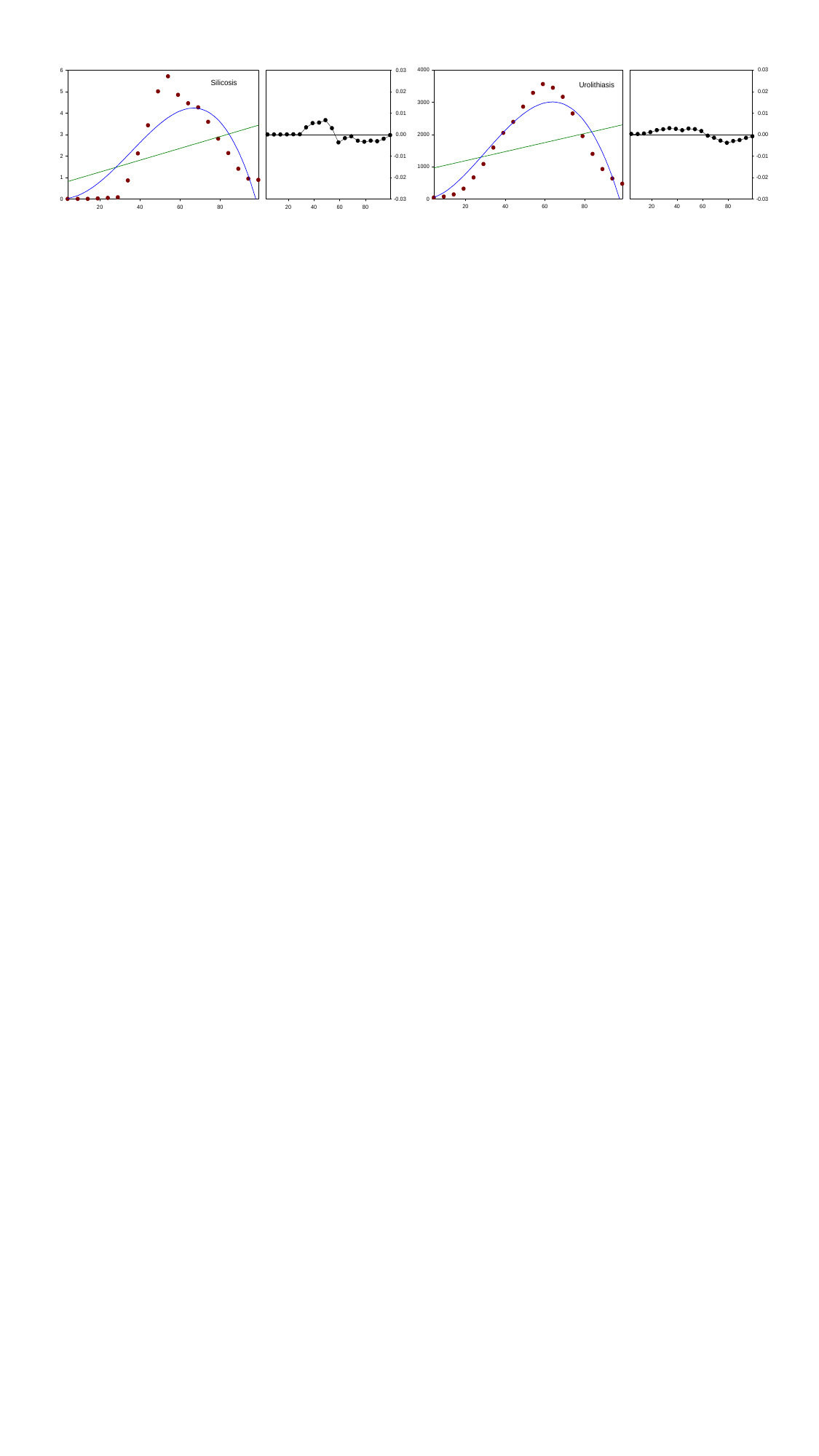

## Slide 11
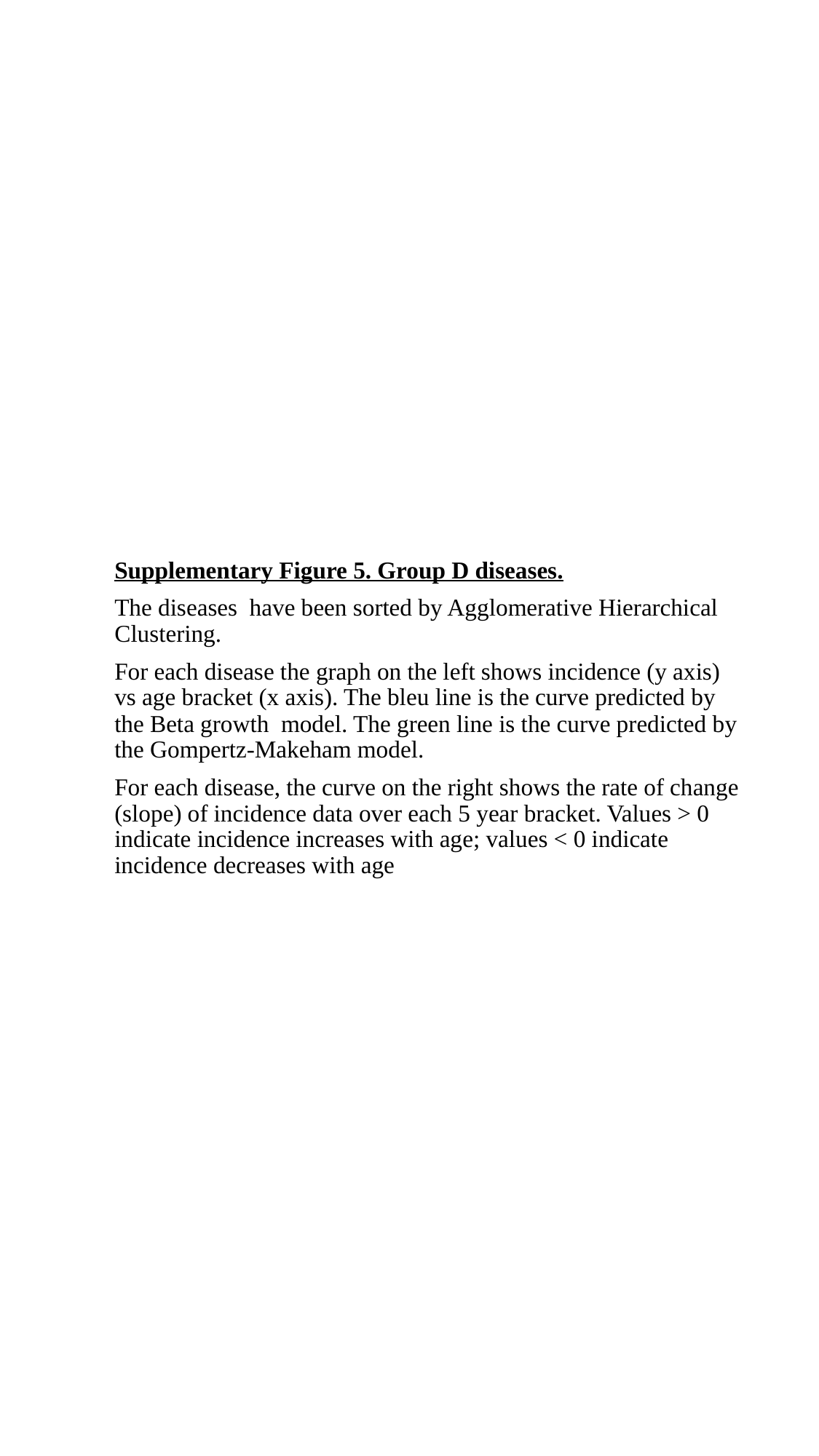

Supplementary Figure 5. Group D diseases.
The diseases have been sorted by Agglomerative Hierarchical Clustering.
For each disease the graph on the left shows incidence (y axis) vs age bracket (x axis). The bleu line is the curve predicted by the Beta growth model. The green line is the curve predicted by the Gompertz-Makeham model.
For each disease, the curve on the right shows the rate of change (slope) of incidence data over each 5 year bracket. Values > 0 indicate incidence increases with age; values < 0 indicate incidence decreases with age

## Slide 12
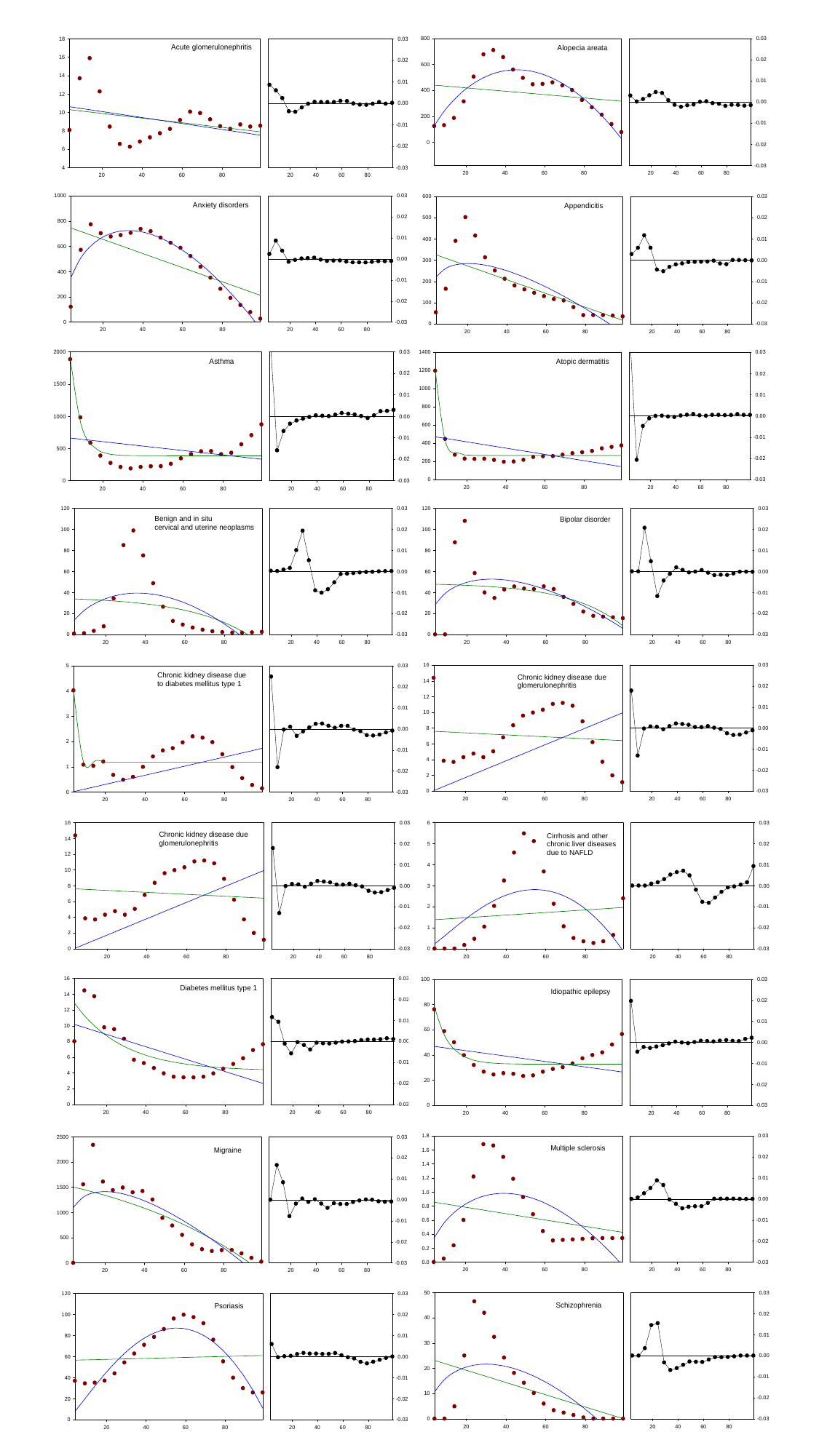

## Slide 13
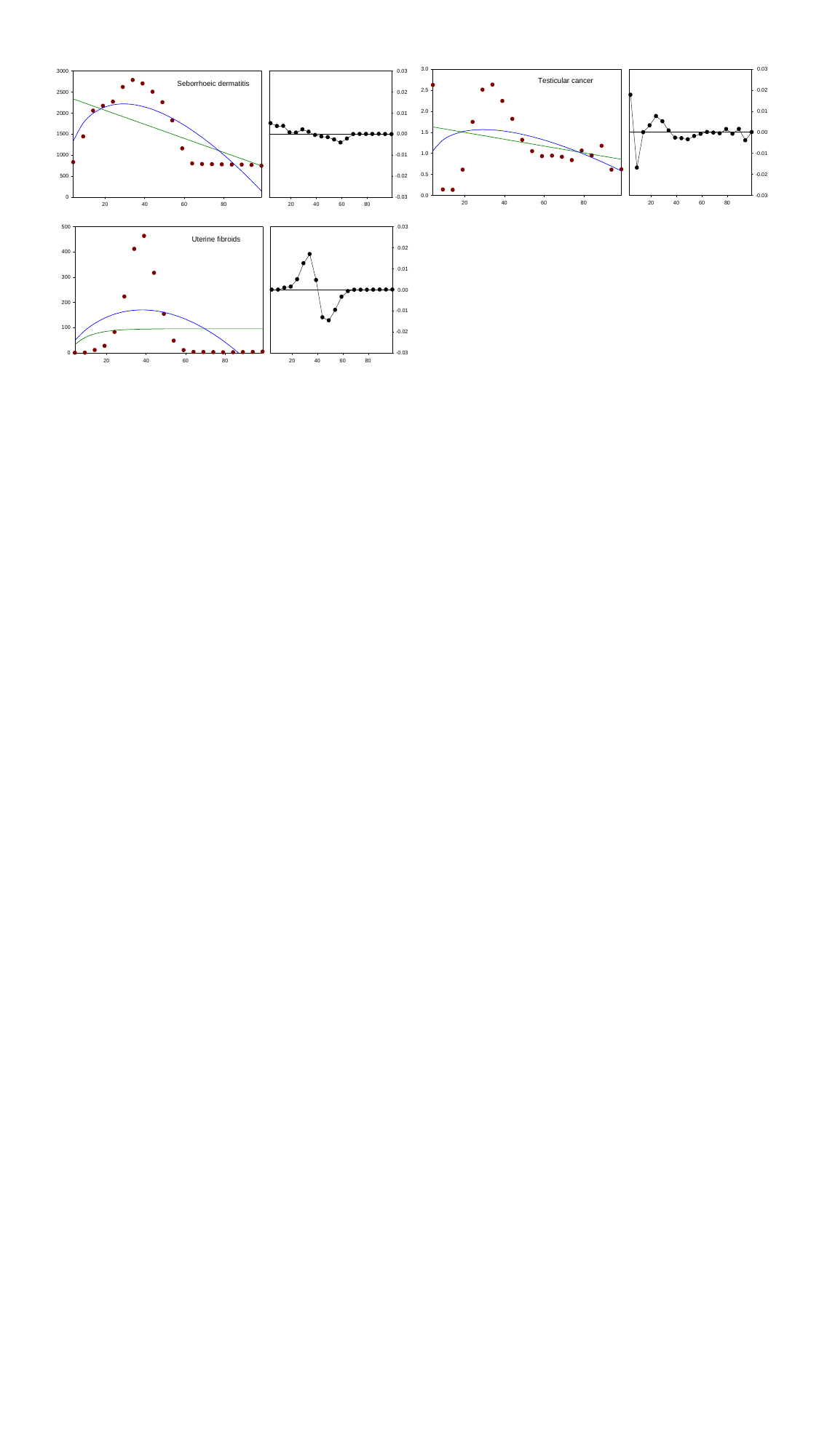

## Slide 14
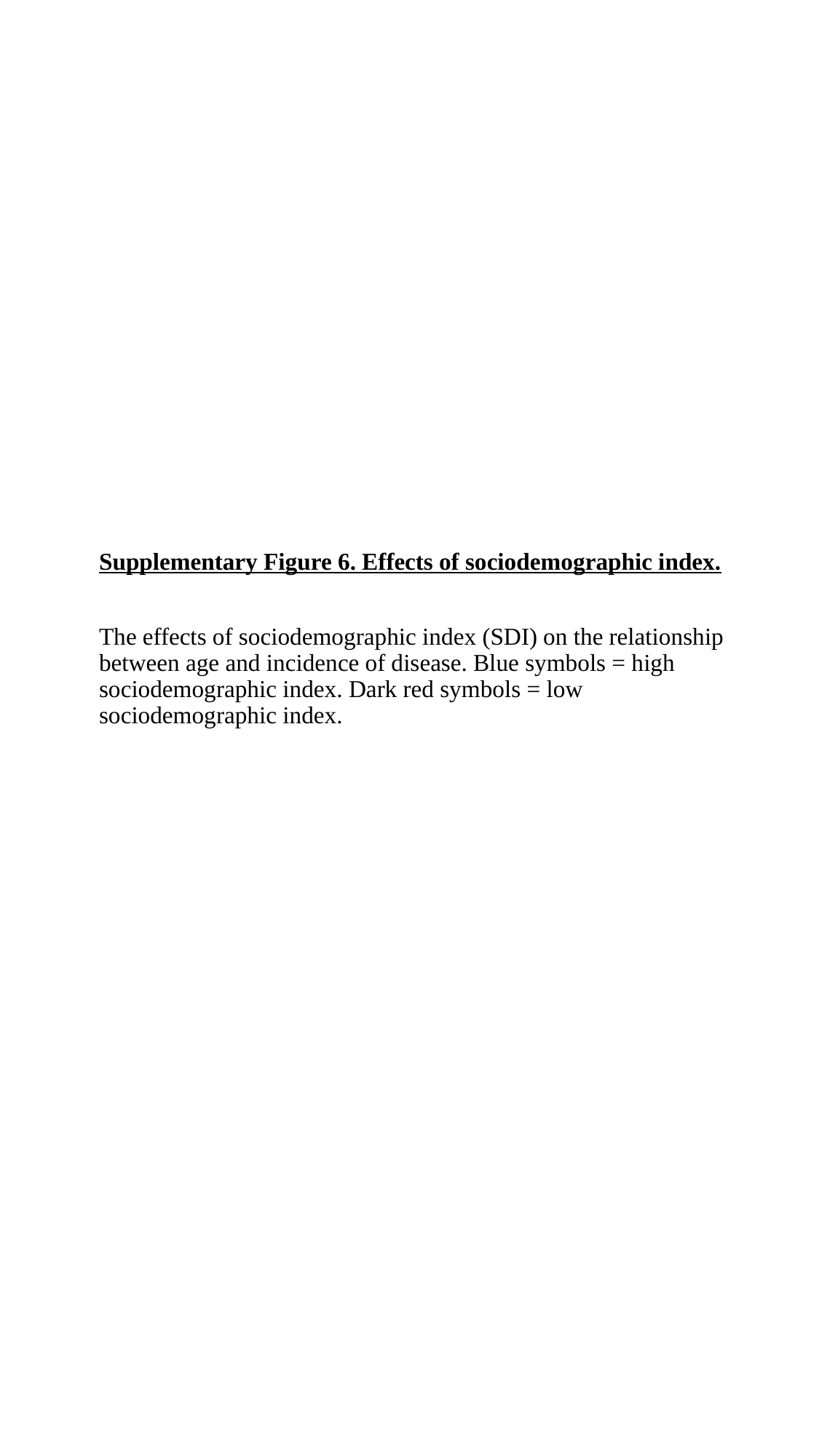

Supplementary Figure 6. Effects of sociodemographic index.
The effects of sociodemographic index (SDI) on the relationship between age and incidence of disease. Blue symbols = high sociodemographic index. Dark red symbols = low sociodemographic index.

## Slide 15
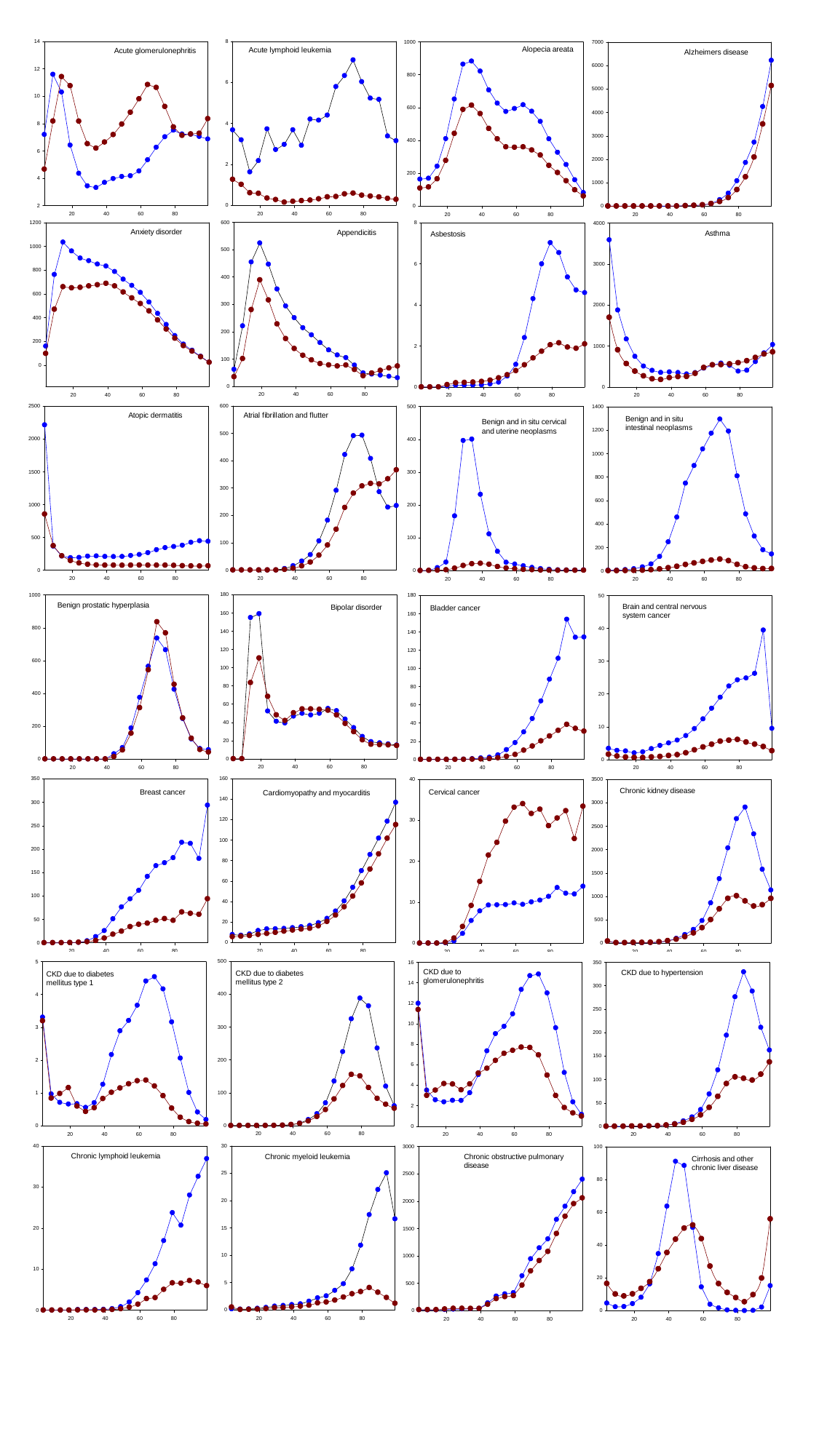

## Slide 16
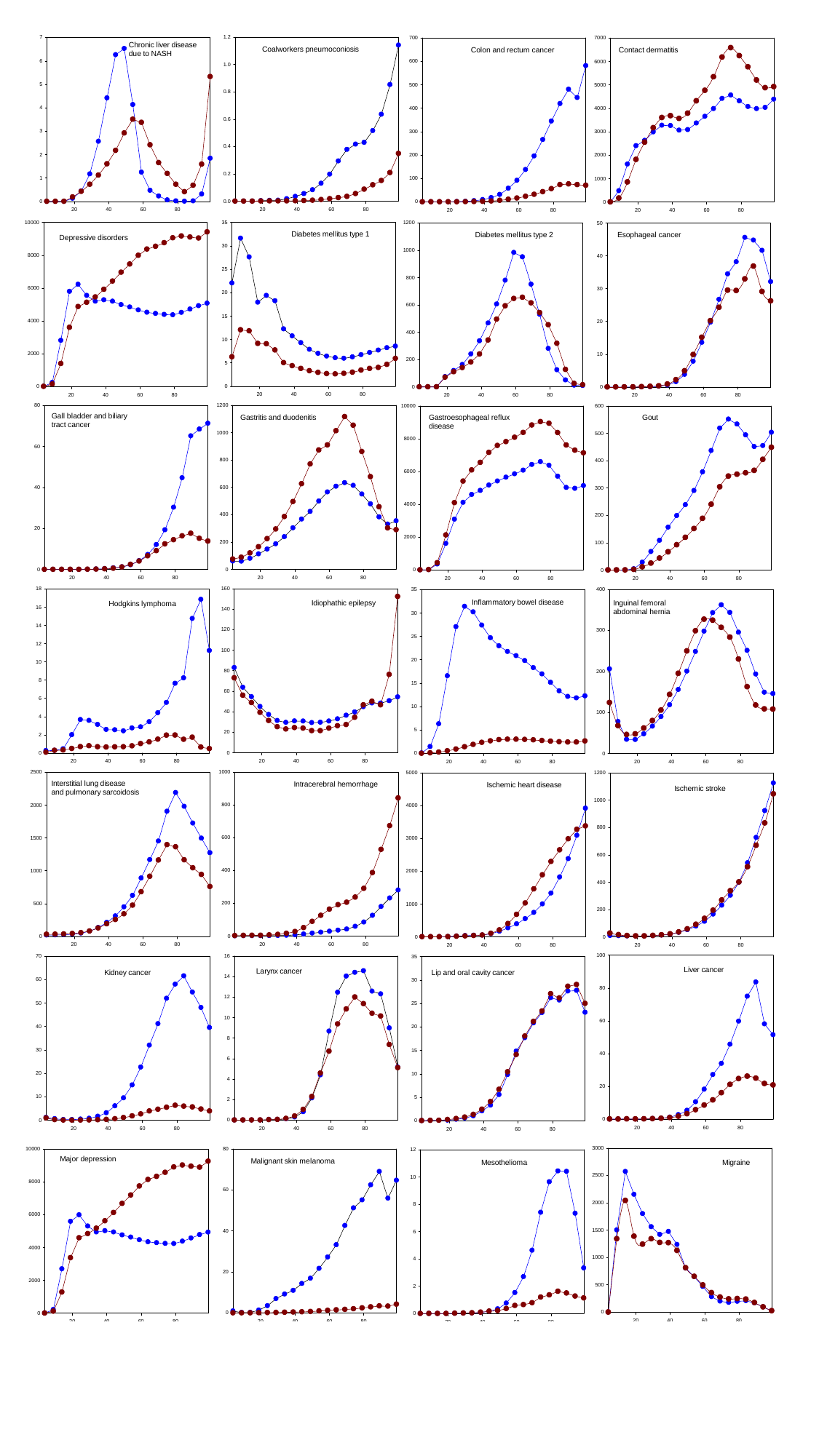

## Slide 17
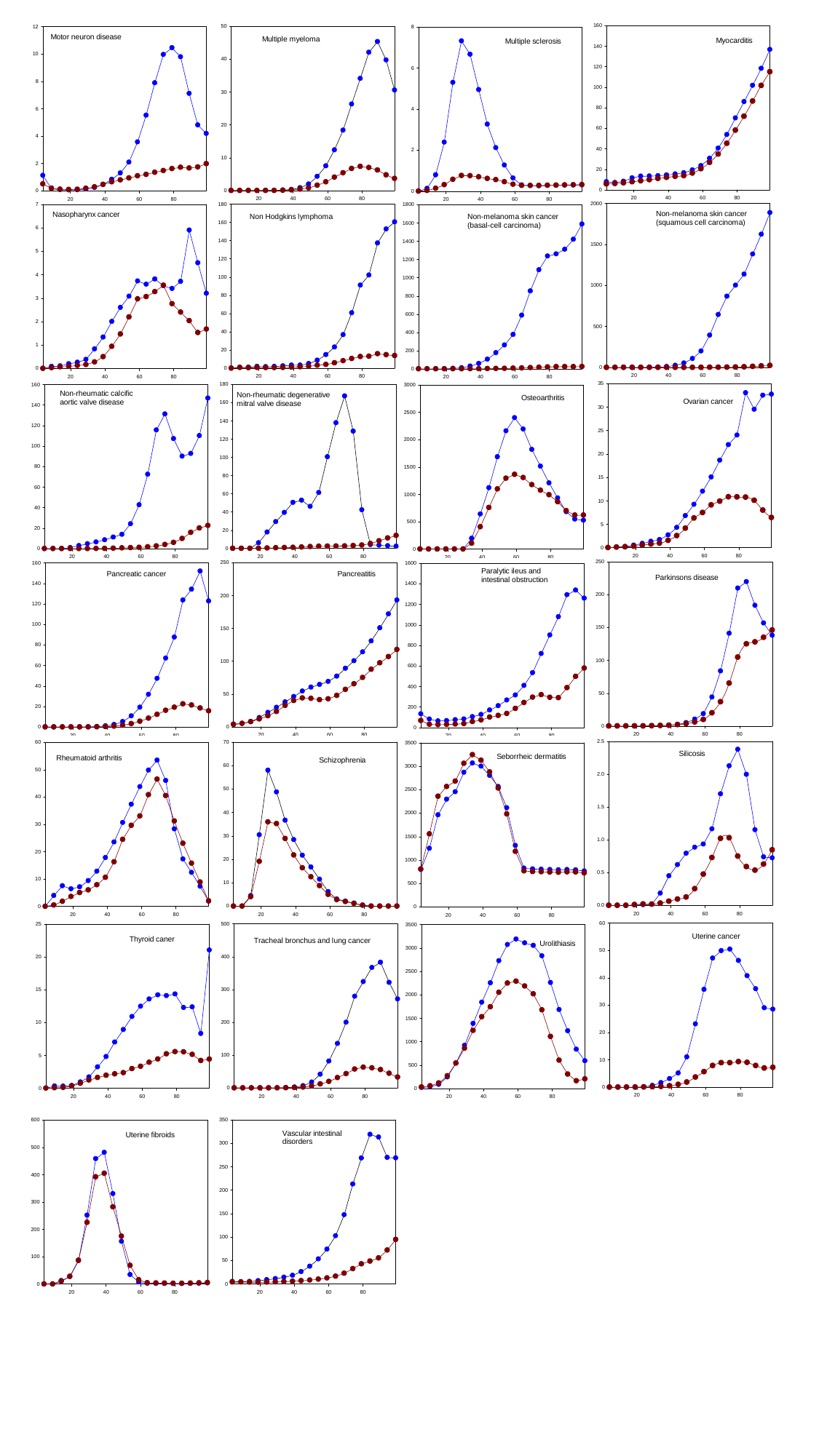

## Slide 18
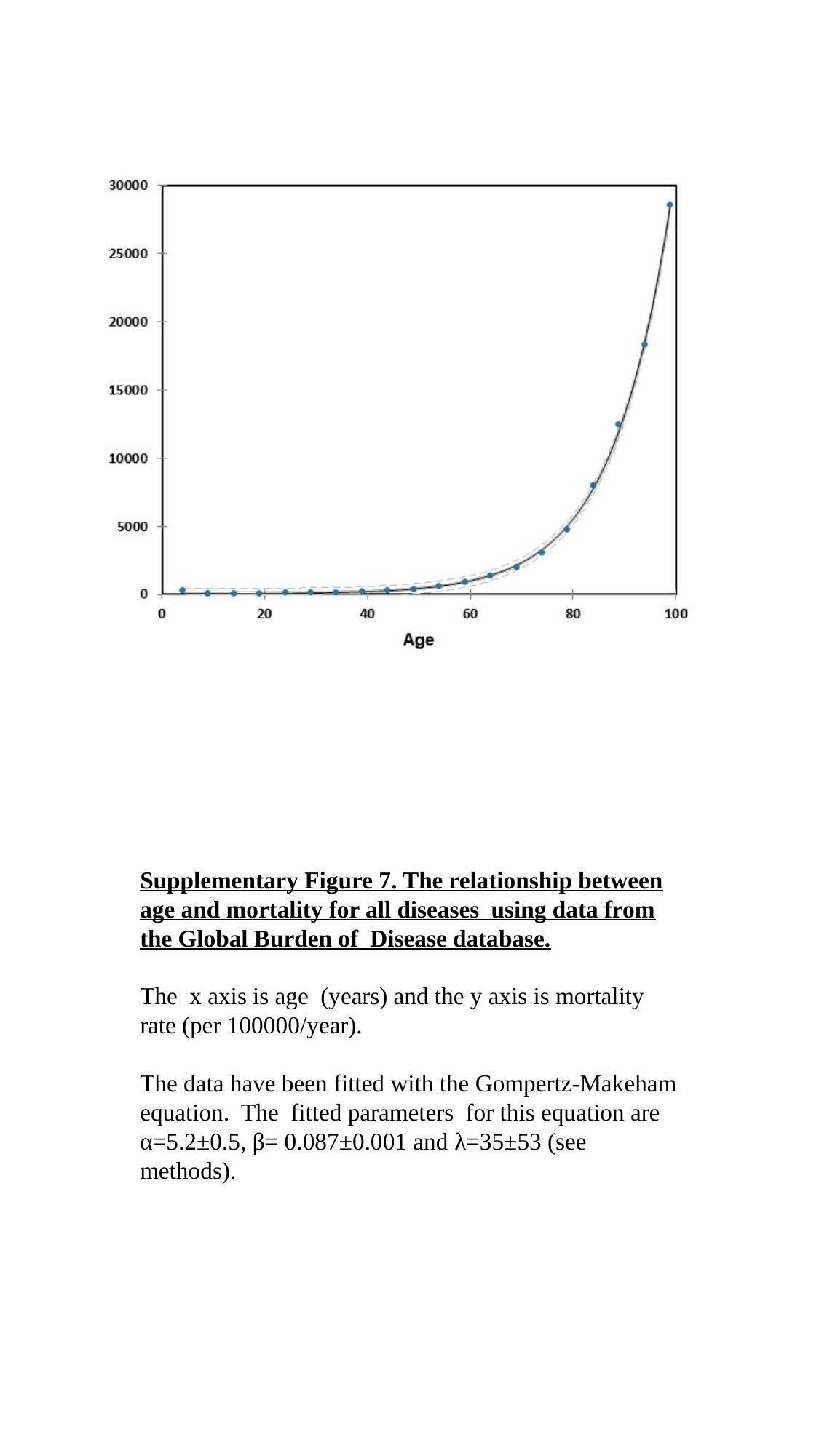

Supplementary Figure 7. The relationship between age and mortality for all diseases using data from the Global Burden of Disease database.
The x axis is age (years) and the y axis is mortality rate (per 100000/year).
The data have been fitted with the Gompertz-Makeham equation. The fitted parameters for this equation are α=5.2±0.5, β= 0.087±0.001 and λ=35±53 (see methods).
